# Almond-like Aroma Formation of Acid Whey by Ischnoderma benzoinum Fermentation: Potential Application in Novel Beverage Development

**DOI:** 10.1021/acs.jafc.5c01359

**Published:** 2025-05-08

**Authors:** Lea Hannemann, Raphaela Klauss, Anne Gleissle, Patrick Heinrich, Thomas Braunbeck, Yanyan Zhang

**Affiliations:** † Department of Flavor Chemistry, Institute of Food Science and Biotechnology, University of Hohenheim, Fruwirthstr. 12, 70599 Stuttgart, Germany; ‡ Department of Soft Matter Science and Dairy Technology, Institute of Food Science and Biotechnology, 26558 University of Hohenheim, Garbenstr. 21, 70599 Stuttgart, Germany; § Aquatic Ecology and Toxicology Group, Center for Organismal Studies, University of Heidelberg, Im Neuenheimer Feld 504, 69120 Heidelberg, Germany

**Keywords:** acid whey, basidiomycota, fermentation, odor-active compounds, cytotoxicity, genotoxicity

## Abstract

To address the sourish off-aroma of acid whey and enhance its upcycling, a new basidiomycete Ischnoderma benzoinum-mediated fermentation system was developed using pure acid whey as the sole substrate. A pleasant sweetish and marzipan-like odor was perceived after fermentation within 7 d at 24 °C in darkness, which was shaped from key contributors including 4-methoxybenzaldehyde (odor activity value (OAV) 878), 3-methylbutanal (OAV 511), 3,4-dimethoxybenzaldehyde (OAV 50), and benzaldehyde (OAV 28). The typical sweetish and almond-like odor persisted well after ultrahigh-temperature processing, though its intensity decreased slightly. Concurrently, the fermentation reduced lactose from 52 to 20 g/L but increased the contents of essential amino acids like threonine, leucine, and lysine. No significant cytotoxicity or genotoxicity differences were found between fermented and unfermented whey. Overall, the study highlights the capability of I. benzoinum fermentation to enhance the flavor of acid whey, offering a promising approach for creating nutritional and flavorful acid-whey-based products.

## Graphical abstract

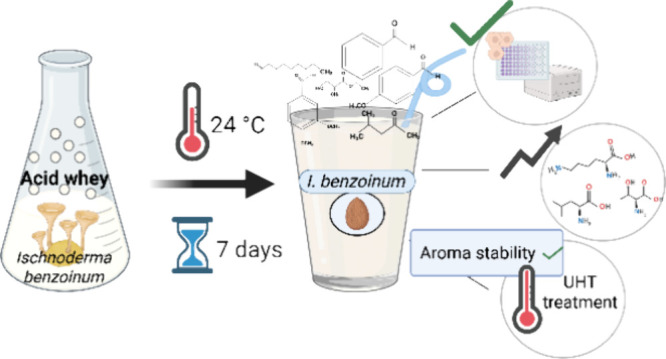

## 1 Introduction

Acid whey (pH < 5.0) is a common byproduct of fresh cheese and certain yogurt manufacturing processes, where acidification is achieved through the activity of lactic acid bacteria or the addition of organic acids like citric and acetic acid.^1^ Despite its high nutritional value, including proteins, vitamins, and minerals, acid whey faces significant challenges in reuse.^2^ In comparison to sweet whey the reusage of acid whey presents some challenges due to its high lactose (35–50 g/L) and mineral (0.5–1.0%) contents, and low acidity (pH < 5).^2–4^ While sweet whey can undergo various purification processes to produce valuable components such as, sweet whey powder, whey protein concentrate,^5^ and can even be used in the production of whey-based beverages, acid whey’s applications are more restricted, primarily to use as sour whey powder in cheese preparations and baked goods.^2^ To overcome these limitations, efforts to upcycle acid whey are focusing on improving lactose separation and isolation, with techniques developed by companies like Danone and Arla Foods Ingredients.^6^ Moreover acid whey is explored for various applications, including anaerobic digestion,^7^ and whey protein isolate extraction,^8^ highlighting its potential for sustainable practices and functional food ingredients. Among these applications, bioethanol production has emerged as a promising sustainable alternative. Yeasts such as Saccharomyces cerevisiae, Candida pseudotropicalis, and Kluyveromyces fragilis are used in the fermentation process, with optimized methods achieving ethanol yields of up to 98%,^9^ highlighting acid whey as a valuable feedstock for bioethanol production. Nevertheless acid whey remains underutilized, forcing industries to dispose this byproduct at high costs, despite its possess valuable content.^3,6^ Acid whey has a high biological and chemical oxygen demand (BOD: 40–60 g/L, COD: 50–80 g/L) making its disposal difficult due to environmental concerns, such as eutrophication of lakes and over fertilization of soils.^3,10^ Currently the common applications of acid whey (∼30%) is for animal feed and as fertilizer due to its high nutrient content,^11^ including nitrogen and phosphorus.^8^ However, its use as a wetting agent must be limited to avoid the risk of salt toxicity in animals and potential damage to animal stalls.^11^ Similarly, proper processing is required to manage its acidic properties and prevent soil acidification when used as a fertilizer.^8^ As a result, there is increasing interest in exploring alternative, full-scale uses for acid whey-based drinks to reduce waste and mitigate environmental impact. To overcome the fermentation-related limitation and satisfy consumer sensory preference Szudera-Kończal et al.^12^ demonstrated that fermenting acid whey with Galactomyces geotrichum at pH 5 and 30 °C, with added galactose, produced a fruity, honey-like aroma. To date, no cost-effective process without additional supplementation to acid whey has been developed that allows its complete reuse. However, upcycling through the use of basidiomycota presents a promising new technology to address this challenge. Basidiomycota, the highest developed class of fungi, have emerged as a promising tool for natural food aromatization due to their unique biocatalytic capabilities.^13,14^ These fungi possess a diverse secretome, including extracellular enzymes allowing them to break down complex organic matter, such as lignin, amino acids, lipids, and carbohydrates.^15,16^ This metabolic versatility enables basidiomycota to synthesize and transform aroma compounds, making them valuable for food processing applications, such as the natural aromatization,^17^ the reduction of undesirable flavors^18^ as well as generating targeted aroma in a case of cheese-like during the fermentation of soybean protein.^19^ Recent studies show basidiomycetes can create complex flavors even from food waste like apple pomace.^15^ Similarly, acid whey is with potential to be transformed through basidiomycota fermentation to develop novel sensory profiles. Thus, we explored cultivating five basidiomycota in pure acid whey to create unique aromas and enable the full reuse of acid whey, potentially as a novel beverage. This approach involved: (i) building up an efficient fermentation process (ii) to evaluate the aroma profile of the fermented acid whey with trained accessors by quantitative descriptive analysis (QDA) to find optimal fermentation time and basidiomycete-substrate combination; (iii) to identify and quantitate aroma-active compounds in the optimal fermented and unfermented acid whey by means of direct immersion-stir bar sorptive extraction (DI-SBSE) followed by gas chromatography–mass spectrometry-olfactometry (GC-MS-O); (iv) comparing the odor profile and the aroma active compounds of the fermented sample after an ultrahigh-temperature treatment. (v) monitoring the nutritional profile of the fermented and unfermented sample and (vi) evaluating the cytotoxicity and genotoxicity of the fermented samples relative to the unfermented sample on the Hep G2 liver cell line using the neutral red retention^20^ and micronucleus assays.^21^

## 2 Materials and Methods

### 2.1 Chemicals and Materials

Acid whey from fresh cheese production was sourced from the Department of Soft Matter Science and Dairy Technology (University of Hohenheim, Stuttgart, Germany) and stored at −20 °C (max. usage 3 months). Ischnoderma benzoinum was obtained from the Institute of Food Chemistry and Biotechnology (Justus-Liebig-University Giessen, Germany). *Trametes versicolor* and Fomitopsis pinicola from German Collection of Microorganisms and Cell Cultures, *Stereum hirsutum* from Friedrich Schiller University Jena (Germany), and Cyclocybe aegerita from Centraal Bureau voor Schimmelcultures (Utrecht, Netherlands). HepG2 cells were provided by the Aquatic Ecology and Toxicology Section at the Centre for Organismal Studies, University of Heidelberg (Germany). Cultivation materials, including agar, malt extract, peptone, and ROTIHistokitt, were purchased from Carl Roth (Karlsruhe, Germany). Cell culture reagents and DAPI (4′,6-diamidino-2-phenylindole) were sourced from Sigma-Aldrich (Deisenhof, Germany), fetal bovine serum from Merck (Darmstadt, Germany), and 96-well microplates from TPP (Trasadingen, Switzerland).

Chemical reagents included (*E*)-2-octen-1-ol (97%), 1-nonanol (99%), 1-octen-3-ol (98%), 2,3-butanedione (99%), 2-decanone (97%), 2-methoxy-phenol (98%), 2-octanol (98%), 2-pentylfuran (98%), 4-methoxy-benzaldehyde (98%), acetoin (96%), decanal (96%), heptanal (97%), methyl 4-methoxybenzoate (99%), and nonanoic acid (97%) from Alfa Aesar (Haverhill, USA); (*E*)-2-octenal (95%), citronellol (95%), ethyl 2-methylbutyrate (99%), and ethyl octanoate (99%) from J&K (Chaoyang District, Beijing, China); 3-methyl-1-butanol (98.5%), benzaldehyde (99.5%), cinnamaldehyde (98%), decanoic acid (98%), ethanol (99.5%), eugenol (98%), hexanoic acid (98%), vanillin (99%), methanol (99%) and octanoic acid (99.5%) from Carl Roth (Karlsruhe, Germany); (*E*)-ethyl cinnamate (98%) from Thermo Fisher (Sindelfingen, Germany); (*E*,*Z*)-2,4-decadienal (99%), ethyl hexanoate (99%), (*Z*)-3-nonen-1-ol (95%), (*E,E*)-2,4-decadienal (99%), (*Z*)-2-undecenal (95%), 2-acetylthiazole (99%) and 3-phenylpropanoic acid (99%) from Acros Organics (Geel, Belgium); acetic acid (99.5%), 1-decanol (99%), 1-octen-3-one (96%), 2-nonanone (99%), methyl benzoate (99%), nonanal (95%), (*E,E*)-2,4-nonadienal (99%), 3-methylbutanal (97%), geraniol (98%), and octanal (99%) from Sigma-Aldrich (Steinheim, Germany); 1-hexanol (99%), dodecanoic acid (98%), hexanal (99%), phenylethyl alcohol (98%), and 3-nonanone (99%) from Merck (Darmstadt, Germany); 3,4-dimethoxybenzaldehyde (98%) and δ-dodecalactone from TCI (Zwijndrecht, Belgium); 1-dodecanol (99%) from Honeywell; (*E,E*)-2,4-octadienal (99%) from Chempur (Karlsruhe, Germany); and heptanoic acid (97%) from Th. Geyer (Renningen, Germany).

A reference standard with saturated alkanes (C_7_–C_40_, 1 mg/mL, dissolved in *n*-hexane) from Merck (Darmstadt, Germany) was used to determine the linear retention indeces (LRI) of the analytes.

### 2.2 Preparation of Preculture and Main Culture of Basidiomycota

I. benzoinum and S. hirsutum were cultured on agar plates with 30 g/L malt extract, 3 g/L peptone, and 15 g/L agar, while T. versicolor, C. aegerita, and F. pinicola were cultured on plates with 20 g/L malt extract and 15 g/L agar. Incubation occurred at 24 °C for 10–14 days until >80% colonization. Mycelium-covered agar (∼1 cm^2^) was transferred to 100 mL of sterile medium in 250 mL flasks containing malt extract peptone medium for I. benzoinum and S. hirsutum, or malt extract medium for the other fungi. The mixture was homogenized at 10,000 rpm for 10 s and incubated at 24 °C for 7 days. After centrifuging and washing, 10 mL preculture was inoculated into 100 mL acid whey and incubated at 24 °C for 7 days, with samples taken every 4–8 h

### 2.3 Sensory Evaluation

After sampling, the culture broth was centrifuged (10 min, 2150 g, 22 °C), and the supernatant was transferred to 20 mL glass vials. Two assessors from the University of Hohenheim evaluated the odor of each fermented liquid whey sample at room temperature. Samples with a promising odor profile were repeated in triplicate to confirm the results of the fermentation. A panel of 10 assessors (6 female, avgerage age 26, nonsmokers) A sensory training was conducted at the Department of Flavor Chemistry, University of Hohenheim using sniffing stick training in triplicate. Assesors who achieved more than 75% accuracy, were allowed to participate in the study. Sniffing sticks were used to define key odor attributes with reference compounds: 2,3-butanedione (buttery), acetoin (buttery,sweetish), acetic acid (pungent), benzaldehyde (almond-like), 4-methoxybenzaldehyde (sweetish,anise-like), decanal (citrus-like,fatty), citronellol (citrus-like), decanoic acid (soapy), vanillin (vanilla,sweetish), eugenol (umami) and hexanal (green), 3-methyl-1-butanol (sweetish). The panel defined odor profiles for the samples using quantitative descriptive analysis (QDA) via orthonasal and retronasal (sip and spit out) perception. Attributes mentioned by ≥70% of the group were ranked on a 0–5 (0 = no; 3 = moderate; 5 = intense odor). The sensory evaluation was carried out in duplicates. For reference, standards included benzaldehyde (almond-like), 1-octen-3-ol (fungal), vanillin (sweetish), ethyl butyrate (fruity), ethyl acetate (solvent-like), maltol (malty), nonanol (musty), acetic acid (sourish), eugenol (umami) and 2-acetylthiazol (cereal-like) within a concentration range of 10–100 μg/L.

### 2.4 Volatile Isolation of Acid Whey Fermented with I. benzoinum by Direct Immersion-Stir Bar Sorptive Extraction (DI-SBSE)

For DI-SBSE, 10 mL of unfermented and fermented acid whey (I. benzoinum, 7 days) were placed in a 20 mL headspace vial with a 10 mm stir bar (0.5 mm PDMS coating) and extracted for 1 h at 25 °C with 1000 rpm on a multi stirrer plate. The stir bar was rinsed, dried, and placed in a thermal desorption unit (TDU, Gerstel) at 40 °C (1 min hold), coupled to a cooled injection system (CIS, Gerstel).The TDU was ramped to 150 °C at 120 °C/min and held again for 10 min in splitless mode. The transfer temperature to the CIS was set at 250 °C. The volatiles were cryo-focused at −100 °C, equilibrated for 1 min, then heated at a rate of 12 °C/s to 220 °C and held for 10 min to release the analytes to the GC column in split mode (1:2).

### 2.5 Gas Chromatography (GC)

For the GC–MS-O analyses, a 7890B gas chromatograph (GC, Agilent, Waldbronn, Germany) equipped with a polar J&W DB-WAXms column (30 m × 0.25 mm ID, 0.25 μm film thickness, Agilent Technologies) or a nonpolar DB-5 column (30 m × 0.25 mm ID, 0.25 μm film thickness, Agilent Technologies), connected to an Agilent 5977 B Inert Plus mass spectrometry detector (MSD, Agilent), equipped with an olfactometry detection port (ODP, Gerstel) was used for aroma analysis. Helium (5.0, Westfalen, Münster, Germany) was used as a carrier gas with a constant flow rate of 1.62 mL/min. The GC oven temperature was held at 40 °C for 3 min, raised with a rate of 5 °C/min to 240 °C, and held again for 5 min. The following GC parameters were applied: septum purge flow, 3 mL/min; inlet temperature: 250 °C; splitless injection; transfer line temperature GC–MS: 250 °C. MSD had the following conditions: in scan mode: solvent delay: 3 min; total ion chromatogram (TIC); scan range: *m*/*z* 38–330; source temperature: 230 °C; quadrupole temperature: 150 °C. The parameter of the olfactory detection port (ODP 3) was set: transfer line temperature: 250 °C; ODP mixing chamber temperature: 150 °C; makeup gas: nitrogen. For the split ratios (SR), SR of TDU was used at a split ratio of 2. Sniffing was performed in triplicate by the assessor. The odors were considered if the assessor perceived the same odor impression twice.

### 2.6 Compound Identification

Odor-active compounds were identified by the characteristic odor, the linear retention indeces (LRI) on a polar (DB-WAXms) and nonpolar (DB-5) column and the mass spectra in comparison with those of authentic standards and data published in literature and MS database (NIST17). Linear retention indeces of the identified compounds were calculated according to the method of Van den Dool.^22^

### 2.7 Semi-Quantitation and Calculation of Odor Activity Values

Aroma compounds in unfermented and fermented acid whey (with I. benzoinum) were quantitated using the internal standard (IS) method with direct immersion-stir bar sorptive extraction (DI-SBSE) on a polar J&W DB-WAXms column. Each 10 mL sample was spiked with 10 μL of IS (cinnamaldehyde 0.6 mg/L) and extracted under consistent DI-SBSE conditions (refer to Section 2.4). Response factors were determined using authentic standards (0.0076–100 mg/L) in 70% ethanol/deionized water, diluted 1:1000 into an acid whey-like solution (7 g/L casein, 1 g/L sunflower oil, 1 g/L salt, 50 g/L lactose) to simulate the matrix. Extraction was carried out under the same conditions as the samples. The odor activity value (OAV) is calculated by dividing the compound′s concentration in the sample by its odor threshold in water from literature. Quantitation was done in two biological replicates, each measured in triplicate (*n* = 6).

### 2.8 Effect of Ultra-High-Temperature Process on the Overall Flavor of Fermented Acid Whey

To evaluate heat stability, acid whey fermented with I. benzoinum for 7 days was heated to 140 °C for 1 min using a tube heating system^23^ (Department of Soft Matter Science and Dairy Technology). A reference liquid with temperature sensors (ALMEMO) ensured accurate holding time. The odor was assessed according to Section 2.3. For taste evaluation, unfermented (autoclaved) and fermented acid whey (UHT-treated) were swirled in the mouth for 5–10 s and spat out. Taste attributes were rated on a 0–5 scale, with 0 indicating no perception and 5 indicating maximum and aroma compounds were semiquantitated as detailed in Section 2.7.

### 2.9 Physicochemical Analyses: Lactose, Protein, Free Amino Acids, Fatty Acids of Unfermented and Fermented Acid Whey

Acid whey and fermented acid whey were analyzed for physicochemical properties including lactose, protein, free amino acids, and fatty acids in triplicate with two biological replicates.

#### 2.9.1 Lactose

Lactose content was measured using HPLC-LT-ELSD. The analysis employed an Agilent 1100 system (degasser, quaternary pump, autosampler, column oven) and an LT-ELSD Sedex 80 detector (Sedere, France) set at 40 °C, Gain 8, Offset 0 mV, Sampling time 10 Hz, and Filter 10 s. The column was a Unison UK-Amino HT 3 μm (250 mm × 3.0 mm) with a Unison UK Amino 3 μm (5 mm × 2.0 mm) precolumn. Samples, from fat-free dairy products, were processed via centrifugation (Amicon Ultra 10k, Merck) and solid-phase extraction (SPE; Chromafix PS-Mix, Macherey-Nagel). The eluent comprised A (acetonitril) and B (aqueous double-distilled water/acetonitrile 70/30), with a gradient of 92% A (0–2 min), 80% A (2–20 min), 92% A (20–23 min), and 92% A (23–25 min) at 70 °C. The injection volume was 20 μL, with a pressure of ∼135 bar and a flow rate of 1 mL/min.

#### 2.9.2 Crude Protein Content

Nitrogen content was measured using the Kjeldahl method, standardized by Commission Regulation (EC) No. 152/2009 III C. The sample was digested with sulfuric acid and a catalyst, alkalized with sodium hydroxide, and the resulting ammonia distilled into sulfuric acid. Excess acid was titrated with sodium hydroxide.

#### 2.9.3 Free Amino Acid Content

Free amino acid content was determined according to Commission Regulation (EC) No. 152/2009 III F, excluding tryptophan. Amino acids were extracted with diluted hydrochloric acid, and nitrogenous macromolecules were precipitated with sulfosalicylic acid and filtered. The pH was adjusted to 2.2, and amino acids were separated via ion exchange chromatography, then quantitated photometrically at 570 nm using ninhydrin.

#### 2.9.4 Bound Amino Acid Content

Amino acid content, excluding tryptophan, was determined following Commission Regulation (EC) No. 152/2009. Tyrosine was measured in unoxidized hydrolysates, while cysteine and methionine were oxidized to cysteic acid and methionine sulfone before hydrolysis. Oxidation was carried out with performic acid and phenol at 0 °C, neutralized by sodium disulfite. Hydrolysis occurred with hydrochloric acid for 23 h, and the pH was adjusted to 2.2. Amino acids were isolated via ion exchange chromatography and quantitated by ninhydrin reaction with photometric detection at 570 nm (440 nm for proline).

#### 2.9.5 Fat Content and Fatty Acid Spectrum

Raw fat content was measured using method VO (EG) No. 152/2009 III H, involving extraction with petroleum ether, distillation, and residue weighing. Fatty acids were methylated and analyzed by gas chromatography (GC) with flame ionization detection (FID) on a J&W DB-23 column (30 m × 0.25 mm i.d., 0.25 μm film thickness). The injection split ratio was 1:40, with helium as the carrier gas and hydrogen (45 mL/min) and synthetic air (450 mL/min) for the FID detector. The oven temperature program was: 100 °C for 1 min, ramping to 120 °C (5 °C/min), 175 °C (9 °C/min), 180 °C (3 °C/min), 200 °C (2 °C/min), 220 °C (1.5 °C/min), and 240 °C (10 °C/min), held at 240 °C for 5 min.

### 2.10 Cytotoxicity and Genotoxicity in HepG2

The cytotoxicity of fermented and unfermented acid whey was assessed in HepG2 cells derived from human hepatoblastoma^24^ using the neutral red assay^20^ which measures dye retention in intact lysosomes. HepG2 cells were cultured in Minimum Essential Medium supplemented with 2 mM sodium pyruvate, 100 U/mL penicillin, 100 μg/mL streptomycin and 10% (v/v) fetal bovine serum at 37 °C and 5% CO_2_ in a humidified cell incubator. Cells were passaged by trypsinization (0.5% trypsin, 0.2% ethylenediaminetetraacetic acid) every 48 h with a 1:4 split ratio; for experiments, HepG2 cells were used up to passage 30. Cultures were tested negative for mycoplasma infection via DAPI (4′,6-diamidino-2-phenylindole) staining.^25^ Cells were seeded into 96-well plates at 10,000 cells per well and incubated for 24 h. Samples were serially diluted in culture medium containing 0.5% ddH_2_O at a 2-fold concentration. Cells were then treated with 100 μL of these dilutions, resulting in final concentrations ranging from 0.2 to 10%. Controls included medium with 0.5% ddH_2_O and 0.1% saponin as a positive control. After 24 h of treatment, the medium was replaced with 0.005% neutral red solution (dissolved in culture media) for 2 h. Cells were then rinsed with phosphate-buffered saline (PBS), and the retained dye was solubilized with 100 μL of neutral red solvent (50% ethanol, 49% ddH_2_O; 1% acetic acid, v/v). Plates were agitated for 30 min at 200 rpm, and absorbance was measured at 540 nm against a reference of 630 nm. Cell viability was calculated as the ratio of optical density at 540 nm to the mean absorbance of the negative control wells. All measurements were run in triplicate, and cytotoxicity was given as NR_80_, the test concentration resulting in a reduction to 80% neutral red retention (≙ Inhibitory concentration_20_ (IC_20_)). To evaluate the genotoxic potential of the samples, the micronucleus assay was conducted according to OECD guideline 487 (OECD, 2023) using the two highest concentrations of the fermented and unfermented acid whey (5 and 10% v/v). Methylmethanesulfonate (MMS) served as a positive control, with concentrations between 500 and 8 μg/mL evaluated in a preliminary cytotoxicity assay as described. Concentrations below the NR50 were further tested in the micronucleus assay, with 35 μg/mL MMS selected as the final positive control. HepG2 cells (50,000 cells/2 mL) were seeded in 6-well plates with sterilized glass slides at the bottom, allowed to attach for 24 h and treated similarly to cytotoxicity testing (24 h), followed by medium removal, PBS rinse, and a 24-h postincubation in fresh culture medium for mitosis and micronucleus formation. After incubation, slides were fixed in methanol (50 and 100%), stained with 100 ng/mL DAPI for 3 min, dehydrated, mounted in RotiHistokitt, and dried for 48 h. Observations were made at a 500× magnification using a Leica Aristoplan fluorescence microscope. 2.000 cells were evaluated following Fenech (2000) criteria from six replicates per sample. The micronucleated cell rate (%) is expressed as the percentage of cells containing micronuclei relative to the total number of cells with normal morphology, excluding those with abnormal nuclei or over three micronuclei.

### 2.11 Data Analysis

Means, standard deviations, spider web plots, bar charts, and statistical analyses were performed using SigmaPlot 12.5 (Systat Software Inc., USA). Paired *t* tests determined statistical significance (*p* < 0.05). Micronucleus rates were compared to negative controls using Welch’s *t* test with significance levels of *p* < 0.05 and 0.01.

## 3 Results and Discussion

### 3.1 Building-Up a Fermentation Process and Sensory Evaluation of Fermented Acid Whey

In the initial phase of the study, five basidiomycete species were selected to improve the aroma profile of acid whey. A successful fermentation system was established with all selected fungi, as each species grew well in the substrate. Unfermented whey retained its characteristic sourish odor, while fermentation for 7 days produced a range of new odor profiles. The odor characteristics of the fermented acid whey after 168 h are summarized in Table 1, showing that the intense sourish odor (intensity 3.0) of unfermented whey was reduced in all samples fermented with the selected basidiomycota. I. benzoinum transformed the whey’s odor into a pleasant, marzipan-like aroma (3.1) with a sweetish note (2.7), offering a more appealing and complex fragrance. In contrast, other fungal species produced interesting odor profiles but with off-odors. Stereum hirsutum resulted in a sourish (1.0) and fruity (0.5) odor with a sourish off-note. T. versicolor imparted a sweetish (1.1) odor, but also a musty off-odor. C. aegerita produced a sourish (1.8) and fermented (1.0) odor, while F. pinicola generated a fermented (1.6) and fruity (0.8) odor. In terms of appearance, the whey fermented with *S. hirsutum* resulted in a milky white-orange appearance, whereas T. versicolor produced a milky brownish liquid. Similarly, C. aegerita also appeared milky white and F. pinicola showed a milky brownish appearance. Fermentation with I. benzoinum clarified the whey, turning it brownish-yellow, while other fungi left the whey cloudy. This suggests I. benzoinum effectively reduced turbidity, likely by breaking down proteins and fats. Despite variations in pH (4–8) or the use of β-galactosidase, I. benzoinum maintained its fermentation time and sensory profile (data not shown), demonstrating adaptability. However, light exposure hindered the development of the characteristic marzipan-like aroma (data not shown), suggesting that it interfered with the metabolic pathways responsible for producing this odor. The aroma profiles of the unfermented and fermented acid whey with I. benzoinum were analyzed by quantitative descriptive analysis to evaluate the effects of fermentation on the aroma of acid whey (Figure 1). Fermentation causes noticeable changes in the four most prominent odor attributes of acid whey. The intensity of sourish odor decreased from 3.0 in acid whey to 1.1 after fermentation, indicating a reduction in some compounds with acidic odor. The intensity of fruity odor increases from 0.6 to 1.7, suggesting that fermentation enhances this characteristic. Similarly, the intensity of almond-like and sweetish odor increased from 0.5 to 3.1 and 1 to 2.7, indicating that fermentation contributes to the intensification of this note.

**Figure 1:**
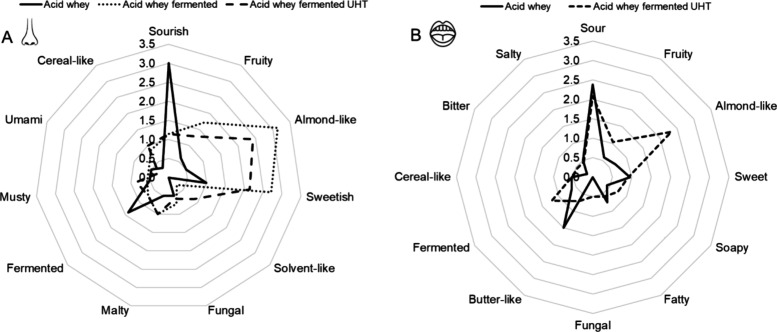
Quantitative descriptive analysis (QDA) of orthonasal perception (A) of unfermented acid whey, acid whey fermented with I. benzoinum, and the UHT-treated fermented acid whey and retronasal perception (B) of the unfermented acid whey and fermented acid whey with I. benzoinum.

**Table 1:** Odor Attribute, Odor Intensity, Appearance, and pH Value of Acid Whey Fermented with Five Fungal Species^a^

		supernatant		
species	fermentation time (h)	odor attribute and odor intensity	appearance	pH
-	-	sourish (3.0)	Milky white	4.5
		fermented (1.7)		
Stereum hirsutum	168	sourish (1.0)	milky white-orange	4.5
		fermented (0.9)		
		fruity (0.5)		
Trametes versicolor	168	fermented (1.5)	milky brownish	4.5
		fruity (1.0)		
		sweetish (1.1)		
Ischnoderma benzoinum	168	marzipan-like (3.1)	clear	4.7
		sweetish (2.7)	brownish-yellowish	
Cyclocybe aegerita	168	sourish (1.8)	milky white	4.6
		fermented (1.0)		
Fomitopsis pinicola	168	fermented (1.6)	milky brownish	4.5
		fruity (0.8)		

a Intensity scale: 0 = no odor perceptible; 3 = moderate odor perceptible; 5 = very intense odor perceptible.

### 3.2 Odorants Comparison of the Unfermented Acid Whey and Fermented Acid Whey with I. benzoinum

To analyze the aroma profile of the supernatants from the acid whey and the fermented acid whey, volatiles were extracted using direct immersion-stir bar sorptive extraction (DI-SBSE) and subsequently analyzed by gas chromatography–mass spectrometry-olfactometry (GC-MS-O) combined with a thermal desorption unit (TDU) and a cooled injection system (CIS) with a polar (DB-WAXms) and nonpolar (DB-5) column. In total 67 aroma compounds could be identified, of which 28 were exclusively found in the unfermented sample (Table 2). These compounds included soapy, fatty, and occasionally buttery compounds, including 2,3-butanedione, which is known for imparting a rich, buttery, or butterscotch-like aroma.^26,27^ Fruity lactones and acetic acid were also detected, with lactones naturally present in milk, contributing to its characteristic aroma, while acetic acid imparts sour, vinegar-like undertones, both aligning with their roles in shaping dairy flavors.^28,29^

**Table 2:** Aroma Compounds Identified in the Unfermented and Fermented Acid Whey with I. benzoinum by Means of DI-SBSE Coupled with GC-MS-O^a^

			LRI						
			DB-WAX column		DB-5 column				
no.	compound	odor impression	sample	standard	sample	standard	identification	unfermented acid whey	fermented acid whey
1	3-methylbutanal	sweetish	908	900			O,LRI,MS,STD		x
2	2,3-butanedione	butter-like	971	972			O,LRI,STD	x	
3	ethyl 2-methylbutyrate	sweetish, fruity	1048	1048	859	851	O,LRI,MS,STD		x
4	methyl 3-methylbutyrate	sweetish, fruity			<800	<800	O,LRI,MS,STD		x
5	hexanal	green	1077	1007	801	803	O,LRI,MS,STD	x	
6	heptanal	citrus-like, fatty	1178	1193	908	903	O,LRI,MS,STD	x	
7	3-methyl-1-butanol	sweetish, malty	1202	1210			O,LRI,,MS,STD		x
8	ethyl hexanoate	sweetish, fruity	1227	1224			O,LRI,MS,STD		x
9	2-pentylfuran	potato-like	1233	1222			O,LRI,MS,STD	x	
10	acetoin	butter-like	1285	1278			O,LRI,MS,STD	x	x
11	1-octen-3-one	fungal	1291	1291	982	978	O,LRI,STD	x	x
12	hexyl propanoate	flowery, citrus-like	1330	1333			O,LRI,STD	x	x
13	1-hexanol	artificial, grassy	1348	1348	874	872	O,LRI,MS,STD	x	
14	2-nonanone	fruity	1387	1380	1099	1095	O,LRI,MS,STD	x	
15	Nonanal	citrus-like, fatty	1388	1384	1106	1104	O,LRI,MS,STD	x	x
16	2-octanol	fruity, soapy	1415	1410	998	994	O,LRI,MS,STD		x
17	(*E*)-2-octenal	fatty, nutty	1419	1420	1063	1059	O,LRI,MS,STD		x
18	ethyl octanoate	citrus-like, green	1423	1426		-	O,LRI,STD		x
19	acetic acid	vinegar-like	1431	1444		-	O, LRI,MS,STD	x	
20	1-octen-3-ol	fungal	1441	1436	984	983	O, LRI,MS,STD	x	
21	n.I	fungal	1476			-	O,LRI	x	
22	2-decanone	fruity, floral	1487	1491		-	O, LRI,MS,STD	x	
23	decanal	fatty, soapy	1497	1491	1207	1204	O, LRI,MS,STD	x	x
24	benzaldehyd	almond-like	1516	1515	967	968	O, LRI,MS,STD		x
25	(*E,E*)-2,4-nonadienal	bell pepper-like, green	1521	1524	1161	1196	O, LRI,STD		x
26	n.I	menthol, herbs	1575	1573			O, LRI	x	
27	(*E,E*)-2,4-octadienal	green, fatty	1584	1587			O, LRI,STD	x	x
28	(*E*)-2-octen-1-ol	fatty, soapy	1605	1609			O, LRI,STD	x	
29	methyl benzoate	Sweetish, fruity	1614	1614			O, LRI,MS		x
30	phenylacetaldehyde	floral, honey-like	1628	1651	1049	1046	O, LRI,MS,STD	x	
31	1-nonanol	fatty, soapy	1650	1646			O, LRI,STD		x
32	(*Z*)-3-nonen-1-ol	fatty, green	1694	1699	1220	1143	O, LRI,MS,STD	x	x
33	n.I	cardboard-like	1714				O, LRI		x
34	(*E,Z*)-2,4-decadienal	green, fatty	1746	1754			O,LRI,STD	x	x
35	(*E,E*)-2,4-decadienal	fatty, deep-fried			1319	1318	O,LRI,MS,STD	x	
36	1-decanol	waxy, citrus-like	1765	1751			O,LRI,STD	x	
37	citronellol	citrus-like	1774	1765			O,LRI,STD	x	x
38	hexanoic acid	fatty, sweaty			1010	1015	O,LRI,STD	x	x
39	2-phenylethyl acetate	sweetish, floral	1803	1807	1251	1255	O,LRI,STD	x	x
40	n.I	fatty, meaty	1857	1815			O,LRI		x
41	geraniol	citrus-like, sweetish	1855	1841	1253	1255	O,LRI,STD		x
42	2-methoxyphenol	smoky	1876	1878	1099	1090	O,LRI,STD		x
43	phenylethyl alcohol	floral, sweetish	1912	1908			O,LRI,STD	x	x
44	1-dodecanol	citrus, soapy	1957	1952	1474	1474	O,LRI,STD	x	x
45	n.I	metallic, algae	2002	2000			O,LRI	x	x
46	4-methoxybenzaldehyde	sweetish, anise-like	2013	2013	1264	1260	O,LRI,MS,STD	x	x
47	octanoic acid	soapy, musty	2053	2052	1184	1183	O,LRI,MS,STD	x	
48	(*E*)-ethyl cinnamate	cinnamon-like	2118	2122	1463	1467	O,LRI,MS,STD	x	x
49	methyl 4-methoxybenzoate	vanilla, sweetish	2088	2092	1382	1386	O,LRI,STD	x	x
50	nonanoic acid	herbal, moldy	2149	2138			O,LRI,MS,STD	x	
51	n.I	cinnamon, phenolic	2180	2177	1169	1162	O,LRI	x	
52	eugenol	pepper, clove-like			1356	1360	O,LRI,MS,STD	x	
53	δ-dodecalactone	coconut-like	2191	2183	1704	1707	O,LRI,STD	x	
54	decanoic acid	fatty, soapy	2258	2246	1376	1374	O,LRI,MS,STD	x	
55	γ-dodecalactone	fruity	2376	2375	1684	1689	O,LRI,STD	x	x
56	3,4-dimethoxybenzaldehyde	sweetish, powdery	2383	2383	1485	1485	O,LRI,MS,STD		x
57	n.I	cirus-like	2395				O,LRI	x	
58	n.I	vanilla, sweetish	2446				O,LRI	x	
59	dodecanoic acid	soapy, waxy	2474	2475	1566	1566	O,LRI,MS,STD	x	
60	dimethyl trisulfide	sulfuric			973	967	O,LRI,STD	x	
61	2-octanol	citrus-like, soapy			998	994	O,LRI,MS,STD		x
62	octanal	citrus-like			1003	1006	O,LRI,MS,STD	x	
63	n.I	fungal			1082	1076	O, LRI		x
64	heptanoic acid	rancid, sweaty			1082	1086	O,LRI,MS,STD	x	
65	3-nonanone	fruity, soapy			1089	1082	O,LRI,MS,STD		x
66	3-phenylpropanoic acid	floral, cinnamon			1335	1337	O,LRI,STD	x	
67	(*Z*)-2-undecenal	mint-like, sweetish			1369	1363	O,LRI,MS,STD		x

a O = odor; (LRI) **=** linear retention index; MS = mass spectra, STD = authentic standard; x = detected; n.I = not identified.

A total of 31 aroma compounds were semiquantitated using the internal standard method (Table 3).

**Table 3:** Concentration (μg/L) and Odor Activity Values (OAVs) of Volatile Compounds in Unfermented and Fermented Acid Whey, as well as Ultra-High-Temperature (UHT) Treated Fermented Acid Whey

		concentration (μg/L)^a^			OAV			
compound	odor threshold (μg/L)	unfermented acid whey	fermented acid whey	UHT treated fermented acid whey	unfermented acid whey	fermented acid whey	UHT treated fermented acid whey	*P*-values Fermented vs UHT
3-methylbutanal	1.1^b^		562 ± 295	240 ± 116		511	219	<0.001
ethyl 2-methylbutyrate	0.8^c^		26 ± 7			41		
hexanal	4.5^d^	140 ± 48			31			
heptanal	3^e^	24 ± 3			8			
3-methyl-1-butanol	250^d^		2350 ± 959	1897 ± 325		9	8	<0.001
ethyl hexanoate	1^d^		31 ± 1			31		
2-pentylfuran	6^d^	66 ± 46			11			
acetoin	800^d^	351,775 ± 17,082	30,564 ± 5584	13,140 ± 1509	440	38	16	
2-nonanone	200^f^	4 ± 1			<1			
nonanal	1^d^	27 ± 5	15 ± 9	30 ± 9	27	15	30	<0.001
2-octanol	9^g^		31 ± 13	24 ± 10		3	3	0.088
(*E*)-2-octenal	3^d^		23 ± 7	19 ± 9		8	6	0.630
Acetic acid	22,000^d^	2,249,551 ± 416,832			102			
furfural	5800^e^	115 ± 50	74 ± 22	1054 ± 383	<1	<1	<1	<0.001
2-decanone	0.19^d^	30 ± 5			157			
Decanal	0.1^d^	4 ± 1	20 ± 3	4 ± 1	40	200	40	<0.001
benzaldehyde	350^d^	237 ± 42	8938 ± 2608	7400 ± 3658	>1	25	21	0.232
methyl benzoate	110^h^		37 ± 11	30 ± 14		1	<1	
1-nonanol	50^d^		90 ± 72	80 ± 45		2	2	0.745
1 -decanol	47^i^		135 ± 87		*x*	3		
1-dodecanol	16^j^	305 ± 104			19			
4-methoxybenzaldehyde	27^k^	609 ± 17	23,700 ± 481	14,490 ± 600	23	878	537	0.002
octanoic acid	3000^d^	8968 ± 2719			3			
(*E*)-ethyl cinnamate	7^d^	87 ± 40	160 ± 50	150 ± 20	12	23	21	0.828
nonanoic acid	3000^d^	150 ± 64			<1			
δ-dodecalactone	100^d^	16 ± 1			<1			
decanoic acid	10,000^e^	16,287 ± 6862			2			
γ-dodecalactone	7^d^	23 ± 9			3			
3,4-dimethoxybenzaldehyde	210^l^		10,513 ± 248	5248 ± 894	-	50	25	0.005
dodecanoic acid	7200^e^	14,516 ± 8046			2			

a Quantitate using semi-quantification with an internal standard.

b Thresholds were obtained from Chen et al.^41^

c Osafune et al.^42^

d Leffingwell & Associates.^43^

e van Gemert.^44^

f Buttery et al.^45^

g Haz-map.com/Agents/3155.^46^

h Scentree.co/en/map/General.^47^

i Ahmed et al.^100^

j Yu et al.^49^

k Ruisinger & Schieberle.^48^

l Murray et al.^50^

This semiquantitation approach allowed us to track relative changes in volatile compounds during fermentation with I. benzoinum. OAVs were calculated using odor thresholds in water to estimate sensory relevance. While not providing absolute quantitation, this method effectively highlights trends and the fermentation’s impact on the aroma profile. Gas chromatography–mass spectrometry-olfactometry (GC-MS-O) analysis identified several key compounds contributing to the aroma profile of unfermented acid whey. These include acetoin (OAV 440), which contributes a fruity odor; 2-decanone (OAV 157), which adds a soapy note; acetic acid (OAV 102), responsible for the sourish, vinegar-like odor; hexanal (OAV 31) and nonanal (OAV 27), both contributing fruity odors; and 1-dodecanol (OAV 19), which imparts a rancid odor. In general, an OAV above 1 indicates a strong flavor contribution, while values below 1 suggest little or no impact.^29^ The sensory profile of the unfermented whey (Figure 1A) was notably sourish, not sweetish or fruity, with the strongest contributors being possibly acetic acid (2245 μg/L) and acetoin (352 μg/L), followed by 2-decanone (30 μg/L) and hexanal (140 μg/L). Decanoic acid (16,287 μg/L) and dodecanoic acid (14,516 μg/L), despite their strong soapy odors, had minimal impact due to high odor thresholds. Most lactones, except γ-dodecalactone, were identified by linear retention index and odor impression but not detected by mass spectrometry. Techniques like linear retention index and gas chromatography-olfactometry (GC-O) help identify trace, odor-active compounds that mass spectrometry may miss due to tiny signal or spectral overlap.^30–32^ Despite limited mass spectrometry detection, lactones significantly contribute to the aroma of acid whey, enhancing its flavor and complexity with fruity to creamy notes, even in trace amounts.^26,33^ These compounds form during the transformation of fatty acids in dairy products, enriching taste and mouthfeel.^27,34^ Attributed to low thresholds, lactones significantly impact flavor due to complex interactions among volatile compounds, with aroma perception varying by concentration.^35,36^

Fermentation with I. benzoinum produced several odor-active compounds absent in the unfermented sample. For example, 3-methylbutanal, showed high concentrations and OAVs (OAV = 511), making it a key aroma contributor. Esters like ethyl 2-methylbutyrate and ethyl hexanoate, detected only in the fermented samples, added fruity notes with high OAVs (41 and 30, respectively). Aldehydes such as benzaldehyde, 4-methoxybenzaldehyde, and 3,4-dimethoxybenzaldehyde, significantly increased after fermentation. Benzaldehyde’s OAV rose from 1 to 30, enhancing its almond-like aroma. 4-methoxybenzaldehyde reached 23,700 μg/L (OAV = 878) and 3,4-dimethoxybenzaldehyde 10,513 μg/L (OAV = 50), contributing anise-like, sweetish notes to the aroma profile. Nonanal, with a waxy, citrusy aroma, had higher OAVs in the fermented sample (OAV 15), indicating increased odor activity. In contrast, acetic acid and 2-decanone, present in the unfermented sample, were undetectable in the fermented sample, likely due to conversion, while acetoin decreased to 30,654 μg/L (OAV 38). Hexanal and heptanal, contributing fresh, green, and fatty aromas, disappeared after fermentation, suggesting metabolism or transformation by I. benzoinum.

Liang et al. (2023) found that soy whey fermentation developed a pleasant almond-like, sweetish aroma within 24 h, with benzaldehyde and 4-methoxybenzaldehyde reaching 1.0 mg/L and 1.1 mg/L, respectively. In acid whey, similar concentrations (1300 and 300 μg/L) were reached after 24 h (data not shown), but the almond-like aroma was less noticeable due to the overpowering acidic odor. This suggests that reducing sourish aroma compounds like acetic acid may be necessary, explaining why acid whey requires a longer fermentation period (7 days) compared to soy whey. Liang et al. proposed that benzaldehyde forms in fungal mycelia through the conversion of l-phenylalanine to phenylpyruvic acid, which undergoes an aminotransferase reaction. This pathway, based on research by Krings, Hinz, and Berger, involves decarboxylation of phenylpyruvic acid to form phenylacetaldehyde, a precursor to benzaldehyde.^37^ Further oxidation of phenylacetaldehyde leads to phenylacetic acid and benzaldehyde. This process is part of the phenylpropanoid pathway, which also produces other aromatic compounds. In *I. resinosum*, the enzyme phenylalanine ammonia-lyase (PAL) converts l-phenylalanine into cinnamic acid, which is reduced to 3-phenylpropanol, showing the fungi’s ability to produce phenyl-based compounds. Research on I. resinosum and I. benzoinum has shown the production of aromatic compounds like benzaldehyde, 4-methoxybenzaldehyde, and 3,4-dimethoxybenzaldehyde.^37–39^ 4-Methoxybenzaldehyde likely forms via benzaldehyde hydroxylation in both species. Wickramasinghe et al. (2019) observed that in *I. resinosum*, 4-methoxybenzaldehyde increased after day 10, and 3,4-dimethoxybenzaldehyde peaked by day 16, while benzaldehyde rose to 4.5 mg/kg by day 16. Isotope labeling revealed benzyl alcohol as a key precursor, with enzymes like aryl-alcohol oxidase and aryl-aldehyde dehydrogenase playing roles in converting alcohols to aldehydes.^38^ Further studies suggested that 4-methoxybenzaldehyde is transformed into 3,4-dimethoxybenzaldehyde through 4-methoxy-3-hydroxybenzaldehyde, highlighting metabolic regulation.^38^ These studies collectively show that the formation of benzaldehyde, 4-methoxybenzaldehyde, and 3,4-dimethoxybenzaldehyde in mushrooms results from complex metabolic pathways involving key intermediates and enzymes. Precursors like benzyl alcohol, benzoic acid, and l-phenylalanine play pivotal roles in these transformations, with potential for optimizing these pathways for commercial applications in flavor and fragrance production.

### 3.3 Impact of Ultra-High-Temperature Processing on Flavor of Fermented Acid Whey

Heating a fermented dairy product to 140 °C for 1 min, known as ultrahigh-temperature (UHT) treatment, kills harmful microorganisms, extends shelf life without refrigeration, and preserves nutrition and flavor stability.^40^ For potential industry food application, aroma stability of fermented acid whey after UHT treatment was evaluated by both sensory evaluation and instrumental aroma analysis. The sensory evaluation of the heated fermented sample (Figure 1A) showed that while the overall aroma profile remained unchanged, with the predominant attributes and absence of off-odors consistent. Nevertheless, there was a noticeable decrease in the intensity of the almond-like odor (2.5) and the sweetish odor (2.3). This suggests that heat treatment likely reduced the concentration of key aroma compounds, lowering intensity without altering the overall aroma profile. Gustatory evaluation by sip and spit out revealed that fermentation reduces sourness slightly (2.4–2.1), and increased fruity (0.6–1.0) and almond-like (0.7–2.3) notes compared to the unfermented sample (Figure 1B). The buttery flavor decreases in the fermented sample (0.7 vs 1.5), while soapy and fungal notes emerge, reaching 0.8 and 0.5, respectively. Sweetness, fatty, and umami flavors show minimal changes, indicating that the fermentation process primarily enhances certain flavors and introduces new sensory attributes while maintaining others. UHT treatment, however, altered the odorants profile in a more complex manner (Table 3). Key aroma compounds such as 3-methylbutanal, 3-methyl-1-butanol, acetoin, nonanal, decanal and 4-methoxybenzaldeyhde showed significant changes in both concentrations and odor activity values (OAVs) after UHT processing. For instance, 3-methylbutanal decreased from 562 μg/L in the fermented sample to 240 μg/L after UHT, with its OAV decreasing from 511 to 219. Similarly, 3-methyl-1-butanol showed a reduction in concentration from 2350 μg/L in the fermented sample to 1897 μg/L after UHT, with its OAV dropping from 9 to 8. Other compounds, such as furfural, showed an increase after UHT from 74 μg/L in the fermented sample to 1054 μg/L in the UHT-treated sample. This increase in furfural, associated with roasted and nutty notes, may be due to Maillard reactions or other thermal processes induced by the UHT treatment. 3,4-Dimethoxybenzaldehyde, absent in unfermented whey, appeared after fermentation (10,513 μg/L, OAV 50) and dropped post-UHT to 5248 μg/L (OAV 25), yet remained a key aroma contributor, highlighting fermentation’s impact on sensory complexity. Fermentation dramatically increases 4-methoxybenzaldehyde levels, from 609 μg/L in unfermented whey (OAV 23) to 23,700 μg/L (OAV 878), far exceeding its odor threshold of 27 μg/L. In UHT-treated fermented whey, its concentration decreases slightly to 14,490 μg/L (OAV 537) whereas no significant change was observed in benzaldehyde. In summary, UHT treatment of fermented acid whey kept the main aroma profile but with reduced intensity of almond-like, sweetish notes. The decrease in aroma compound could be attributed to thermal degradation, evaporation, and interactions with the whey matrix.

### 3.4 Nutritional-Relevant Physicochemical Analysis of Acid Whey Fermentation Mediated by I. benzoinum

Fermentation of acid whey with I. benzoinum significantly altered its composition. The lactose content decreased significantly from 52.6 ± 10.8 to 20.0 ± 3.6 g/L (Figure 2A), indicating its effective utilization by the fungus. This reduction was reflected by the clear appearance of the whey postfermentation, suggesting efficient lactose metabolism by the fungus. Crude protein content also declined significantly, from 1.3 ± 0.04 to 0.6 ± 0.02%. Moreover, fermentation of acid whey led to significant changes in amino acid concentrations (Figure 2B). For instance, asparagine increased from 29.0 ± 10.8 μg/mL to 51.4 ± 2.4 μg/mL (*p* = 0.027). Other amino acids, including glycine, alanine, valine, isoleucine, leucine, l-phenylalanine, histidine, lysine increased significantly (*p* = 0.001 to <0.001). Interestingly, l-phenylalanine levels increased despite the formation of benzaldehyde, which is known to be derived from this amino acid.^37^ This suggests that the fungus converts l-phenylalanine into benzaldehyde, while simultaneously breaking down proteins to replenish l-phenylalanine levels, leading to an overall increase in its concentration. In contrast, cystine, tyrosine, proline, and taurine showed no significant changes (p = 0.08 to 0.748), indicating minimal effects of fermentation on these amino acids. The raw fat content of the whey remained stable at 0.01%, while fermentation with *I. benzoinum* enriched it with long-chain and polyunsaturated fatty acids. Notably, stearic acid (C18:0) increased from 1.5 to 22.3%, and linoleic acid (C18:2n6c) levels were also elevated (Supporting Information Table S2).

**Figure 2:**
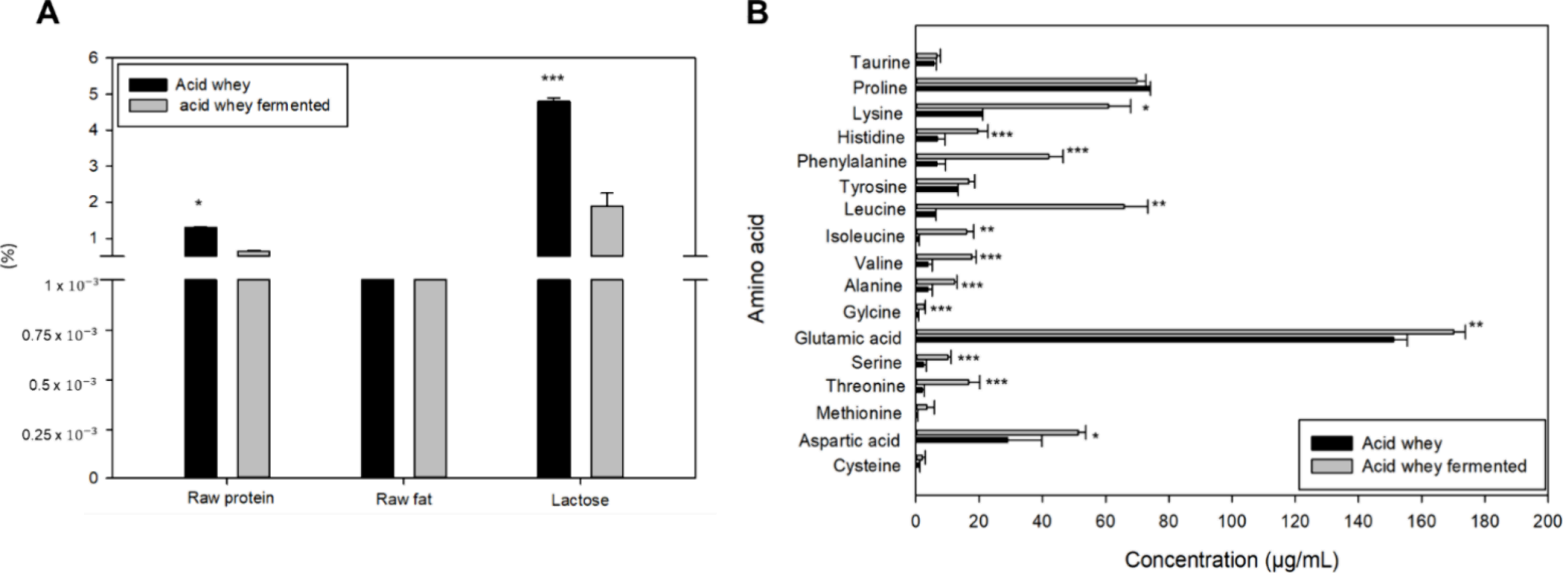
Physicochemical analysis of acid whey and fermented acid whey with I. benzoinum for 7 days: Lactose content, raw fat and raw protein (A) as well as amino acid profile (B). *p*-Values indicated as follows: *p* < 0.05 (*), *p* < 0.01 (**), *p* < 0.001 (***).

### 3.5 Cytotoxicity and Genotoxicity Assay of I. benzoinum Mediated Fermentation of Acid Whey

Cytotoxicity effects of acid whey fermented with I. benzoinum on Hep G2 cells were evaluated using the neutral red assay to assess cell viability and potential toxic effects. Additionally, the micronucleus assay was utilized to detect potential genetic damage and chromosomal abnormalities, providing a comprehensive understanding of the genotoxic risks associated with the fermentation process. This assay combination was selected to provide an initial assessment of the safety of the fermented whey for potential applications. Given the lack of studies on effects by harmful compounds produced during fermentation by I. benzoinum, the neutral red assay assessed potential safety concerns on cell viability and cytotoxicity. Hep G2 cell viability was reduced to <80% when exposed to both the control and the fermented acid whey treated with I. benzoinum, particularly at a concentration of 10% with NR_80_ values of 9.5 ± 0.1 (acid whey) and 8.8 ± 3.1 (acid whey fermented), respectively (Figure 3). The functionality of the assay was validated by negative and positive controls. Notably, achieving a 10% whey concentration in the bloodstream is biologically irrelevant, as whey is metabolized and absorbed in the digestive tract, not directly entering the bloodstream in high volumes to contact hepatocytes, which would correspond to the exposure scenario prevalent in this assay. In addition, the presence of fat and proteins in the acid whey may contribute to cellular stress and an immune response, further reducing cell viability. The lipophilic fats could potentially alter membrane integrity, reducing dye retention and, consequently, reducing cell viability. However, microscopic control revealed active proliferation and healthy cell behavior, indicating maintained functionality despite reduced viability.

**Figure 3:**
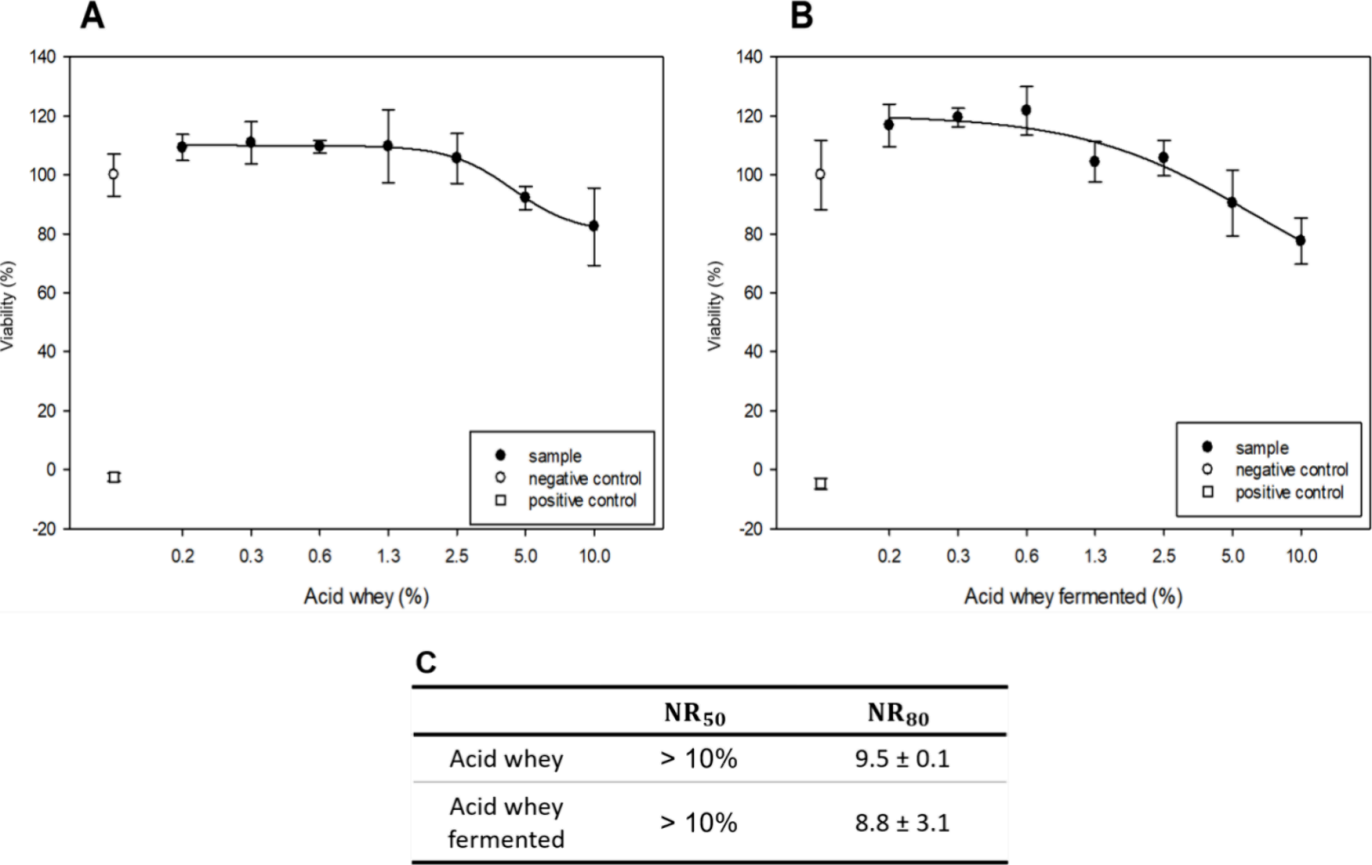
Cell viability of hepatocytes (HepG2) exposed to unfermented acid whey (A) and fermented acid whey with I. benzoinum for 7 days (B). This data represents one run out of three independent trials. The cells were exposed to serial dilutions of the samples, ranging from 0.1 to 10%, for 24 h. Cell culture media served as the negative control, while 0.1% saponin was used as the positive control. Cell viability was assessed using the neutral red assay, which measures lysosomal dye retention. (C) Displays the mean NR_50_ and NR_80_ values from all three replicates, along with their standard deviation.

The micronucleus assay showed low micronucleus rates (0.06–0.12%) for both acid whey and fermented acid whey, comparable to the negative control. Statistical analysis revealed no significant differences between test samples and negative control (Figure 4). The low rates, including those in the positive control, are likely due to the high biotransformation capacity of HepG2 cells, which efficiently detoxify harmful compounds, aligning with findings from Guo et al.^52^ These results indicate that the fermentation with I. benzoinum did not cause chromosomal damage to HepG2 cells. Overall, while the fermented acid whey exhibited some cytotoxicity most probably due to the treatment scenario as discussed, genotoxic effects were minimal, and cells remained functional, being providing no reasonable evidence for significant in vitro genotoxicity or cytotoxicity, when used as a comestible. Still, further studies with more complex test systems (e.g., feeding experiments) are needed to assess possible long-term effects and the safety profile under various conditions.

**Figure 4:**
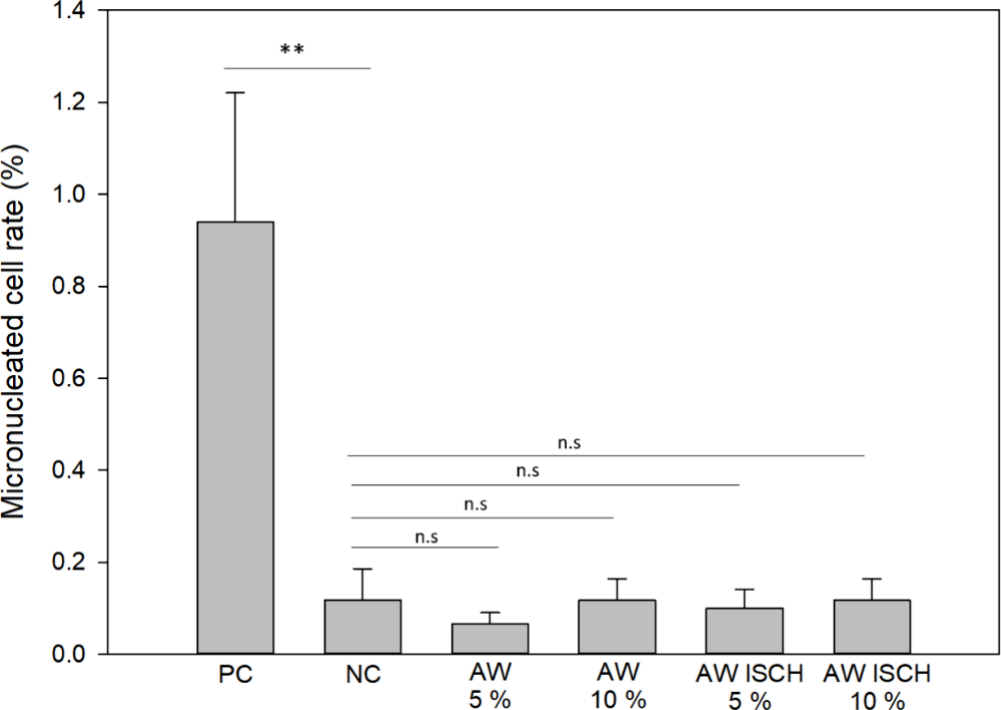
HepG2 cells were exposed to 5 and 10% and of unfermented acid whey (AW) and fermented acid whey with I. benzoinum (AW ISCH) for 24 h. Micronucleated cell rate (%) is presented as the percentage of micronucleated cells over cells with normal morphology. 2.000 cells were evaluated from six replicates per sample. 35 μg/mL of methylmethanesulfonate served as a positive control (PC), while culture medium was used as a negative control (NC). Samples were tested for statistical significance vs NC using Welch’s *t* test with significance levels of *p* < 0.05 and *p* < 0.01 (**).

In conclusion, acid whey proved to be a suitable substrate for all basidiomycota tested, with I. benzoinum showing the best results in enhancing the aroma profile—producing a sweetish, almond-like fragrance while reducing the unappealing sourish odor of unfermented whey. Interesting key aroma compounds like 4-methoxybenzaldehyde (23,700 μg/L), benzaldehyde (8938 μg/L) and 3-methylbutanal (562 μg/L) were formed during fermentation. Although ultrahigh-temperature treatment preserved key flavor profiles, it resulted in a reduction of the almond-like and sweetish odor intensities, which was consistent with quantitative key odorants changes. Importantly, I. benzoinum fermentation led to a significant reduction in lactose (from 52.5 to 20.0 g/L) and increased levels of essential amino acids such as threonine (2–17 μg/mL), leucine (6–66 μg/mL), and lysine (21–61 μg/mL), suggesting an improved nutritional profile. Cytotoxicity and genotoxicity assessments of fermented acid whey showed no significant difference to the unfermented acid whey. These tests provide a preliminary indication of the product’s safety, suggesting that the fermentation process did not produce any harmful compounds, thereby preserving the safety of the fermented product for potential consumption. Compared to previous studies requiring supplementation (e.g., galactose in G. geotrichum fermentations by Szudera-Kończal et al.), our approach offers a cost-effective, supplementation-free method. Unlike conventional uses like animal feed or fertilizer, this study presents a sustainable alternative transforming acid whey into a flavorful beverage with improved sensory and nutritional qualities. I. benzoinum fermentation thus emerges as a viable, scalable solution for acid whey valorization.

## Supplementary Material



## Notes

Mean values and standard deviation of the orthonasal and retronasal sensory evaluation; fatty acid composition of acid whey and acid whey fermented; and cell vitality of hepatocytes (HepG2) exposed to methylmethanesulfonate (PDF)
