# Supplementary material for: Almond-like Aroma Formation of Acid Whey by Ischnoderma benzoinum Fermentation: Potential Application in Novel Beverage Development
Source: J Agric Food Chem. 2025 May 8;73(20):12433–44. doi: 10.1021/acs.jafc.5c01359 (PMC12100700; doi:10.1021/acs.jafc.5c01359)
Supplement: Supplementary file 1 [file jf5c01359_si_001.pdf]

Supporting Information

Almond-Like Aroma Formation of Acid Whey by *Ischnoderma benzoinum* Fermentation: Potential Application in Novel Beverage Development

Lea Hannemann[1], Raphaela Klauss[2], Anne Gleissle[2], Patrick Heinrich[3], Thomas Braunbeck[3], Yanyan ZHANG[1*]

[1] Department of Flavor Chemistry, Institute of Food Science and Biotechnology, University of Hohenheim, Fruwirthstr. 12, 70599 Stuttgart, Germany

[2] Department of Soft Matter Science and Dairy Technology, Institute of Food Science and Biotechnology, University of Hohenheim, Garbenstr. 21, 70599 Stuttgart, Germany

[3] Aquatic Ecology and Toxicology Group, Center for Organismal Studies, University of Heidelberg, Im Neuenheimer Feld 504, 69120, Heidelberg, Germany

Lea Hannemann, Institute of Food Science and Biotechnology, Department of Flavor Chemistry, University of Hohenheim, Stuttgart 70599, Germany. Phone: +49 711459-24872, Email: lea.hannemann@uni-hohenheim.de

Raphaela Klauss, Institute of Food Science and Biotechnology, Department of Soft Matter Science and Dairy Technology, University of Hohenheim, Stuttgart 70599, Germany. +49 711 459-23624, Email: raphaela.klauss@uni-hohenheim.de

Anne Gleissle, Institute of Food Science and Biotechnology, Department of Soft Matter Science and Dairy Technology, University of Hohenheim, Stuttgart 70599, Germany. Phone: +49 711 459-23913, Email: anne.gleissle@uni-hohenheim.de.

Dr. Patrick Heinrich, Aquatic Ecology and Toxicology, Center for Organismal Studies, University of Heidelberg,69117 Heidelberg, Phone: +49 6221/54-6260, Email: patrick.heinrich@cos.uni-heidelberg.de

Prof. Dr. Thomas Braunbeck, Aquatic Ecology and Toxicology, Center for Organismal Studies, University of Heidelberg, 69117 Heidelberg, Phone: +49 6221/54-5668, Email: braunbeck@uni-hd.de

Prof. Dr. Yanyan Zhang, Institute of Food Science and Biotechnology, Department of Flavor Chemistry, University of Hohenheim, Stuttgart 70599, Germany; Tel: +49 711459-24871, Fax: +49 711459-24873, *E-mail: yanyan.zhang@uni-hohenheim.de

* Corresponding author: Prof. Dr. Yanyan Zhang, yanyan.zhang@uni-hohenheim.de; +49-711-459-24871

Table of Content

Table S1 Mean values and standard deviation of quantitative descriptive analysis (QDA) of orthonasal perception of unfermented acid whey, acid whey fermented with *I. benzoinum*, and the UHT-treated fermented acid whey and retronasal perception of the unfermented acid whey and fermented acid whey with *I. benzoinum* (n=10)**...3**

Table S2 Fatty Acid Composition of Acid Whey and Acid Whey Fermented with *Ischnoderma benzoinum* **...4**

Figure S3 Cell vitality of hepatocytes (HepG2) exposed to methyl methane sulfonate (MMS). The cells were exposed to serial dilutions of the samples, ranging from 500 µg/mL to 7.8 µg/mL. Cell culture media served as the negative control. Cell vitality was assessed using the neutral red assay, which measures lysosomal dye retention...**6**

Table S1 Mean values and standard deviation of quantitative descriptive analysis (QDA) of orthonasal perception of unfermented acid whey, acid whey fermented with *I. benzoinum*, and the UHT-treated fermented acid whey and retronasal perception of the unfermented acid whey and fermented acid whey with *I. benzoinum* (n=10).

**Orthonasal**					
Acid whey		Acid whey fermented		Acid whey fermented UHT	
Attribute	Intensity	Attribute	Intensity	Attribute	Intensity
sourish	3.0 ± 0.4	sourish	1.3 ± 0.4	sourish	1.3 ± 0.9
fruity	0.6 ± 0.4	fruity	1.2 ± 0.4	fruity	0.5 ± 0.5
almond-like	0.5 ± 0.3	almond-like	2.8 ± 0.8	almond-like	1.7 ± 0.7
sweetish	1.0 ± 0.7	sweetish	2.2 ± 0.7	sweetish	1.1 ± 0.9
solvent-like	0.0 ± 0.0	solvent-like	0.8 ± 0.6	solvent-like	0.2 ± 0.3
fungal	0.6 ± 0.4	fungal	0.2 ± 0.3	fungal	1.0 ± 0.7
malty	0.4 ± 0.4	malty	1.3 ± 0.9	malty	1.6 ± 1.4
fermented	1.4 ± 0.7	fermented	1.2 ± 0.5	fermented	1.2 ± 0.8
musty	0.5 ± 0.7	musty	0.7 ± 0.6	musty	0.8 ± 0.6
umami	0.5 ± 0.5	umami	0.1 ± 0.3	umami	0.2 ± 0.1
cereal-like	0.2 ± 0.3	cereal-like	0.5 ± 0.5	cereal-like	1.2 ± 0.7
**Retronasal**					
Acid whey		Acid whey fermented UHT			
Attribute	Intensity	Attribute	Intensity		
sour	2.4 ± 0.9	sour	2.1 ± 1.1		
fruity	0.5 ± 0.3	fruity	1.0 ± 0.6		
almond-like	0.7 ± 0.2	almond-like	2.3 ± 0.6		

sweet	1.0 ± 0.8	sweet	0.9 ± 0.3
soapy	0.4 ± 0.1	soapy	0.8 ± 0.2
fatty	0.8 ± 1.2	fatty	0.6 ± 1.0
fungal	0.0 ± 0.0	fungal	0.5 ± 0.6
butter-like	1.5 ± 0.4	butter-like	0.7 ± 0.2
fermented	0.6 ± 1.2	fermented	1.2 ± 1.0
musty	0.2 ± 0.4	musty	0.3 ± 0.6
umami	0.3 ± 0.1	umami	0.5 ± 0.3
cereal-like	0.5 ± 0.7	cereal-like	0.5 ± 0.3
bitter	0.2 ± 0.3	bitter	0.5 ± 0.7
salty	0.5 ± 0.7	salty	0.5 ± 0.4

Table S2 Fatty Acid Composition of Acid Whey and Acid Whey Fermented with *Ischnoderma benzoinum*

	Acid whey [%]	Acid whey fermented with *I. benzoinum* [%]
**C4:0**	2.7 ± 0.5	2.0 ± 1.5
**C6:0**	0.7 ± 0.1	0.2 ± 0.03
**C8:0**	0.5 ± 0.1	0.3 ± 0.1
**C10:0**	1.1 ± 0.1	0.3 ± 0.1
**C12:0**	1.9 ± 0.1	1.3 ± 0.4
**C13:0**	0.2 ± 0.1	0.2 ± 0.1
**C14:0**	5.8 ± 0.4	2.4 ± 0.2
**C14:1**	0.3 ± 0.1	0.4 ± 0.3

**C15:0**	0.9 ± 0.1	0.8 ± 0.1
**C16:0**	28.5 ± 1.9	32.3 ± 3.2
**C16:1**	0.3 ± 0.1	-
**C17:0**	0.9 ± 0.08	1.0 ± 0.1
**C18:0**	1.5 ± 0.3	22.3 ± 2.8
**C18:1n9c**	1.5 ± 0.3	7.3 ± 1.9
**C18:1n9t**	1.5 ± 0.3	0.2 ± 0.1
**C18:2n6c**	2.6 ± 0.8	5.8 ± 0.3
**C18:3n3**	0.4 ± 0.1	0.5 ± 0.3
**C20:0**	0.9 ±0.1	1.4 ± 0.2
**C20:1**	0.2 ± 0.1	-
**C21:0**	0.7 ± 0.1	0.94 ± 0.3
**C22:0**	5.3 ± 0.9	7.08 ± 0.83
**C22:2**	0.7 ± 0.3	-
**C23:0**	4.4 ± 0.5	5.7 ± 0.5
**C24:0**	4.2 ± 0.6	7.6 ± 0.3

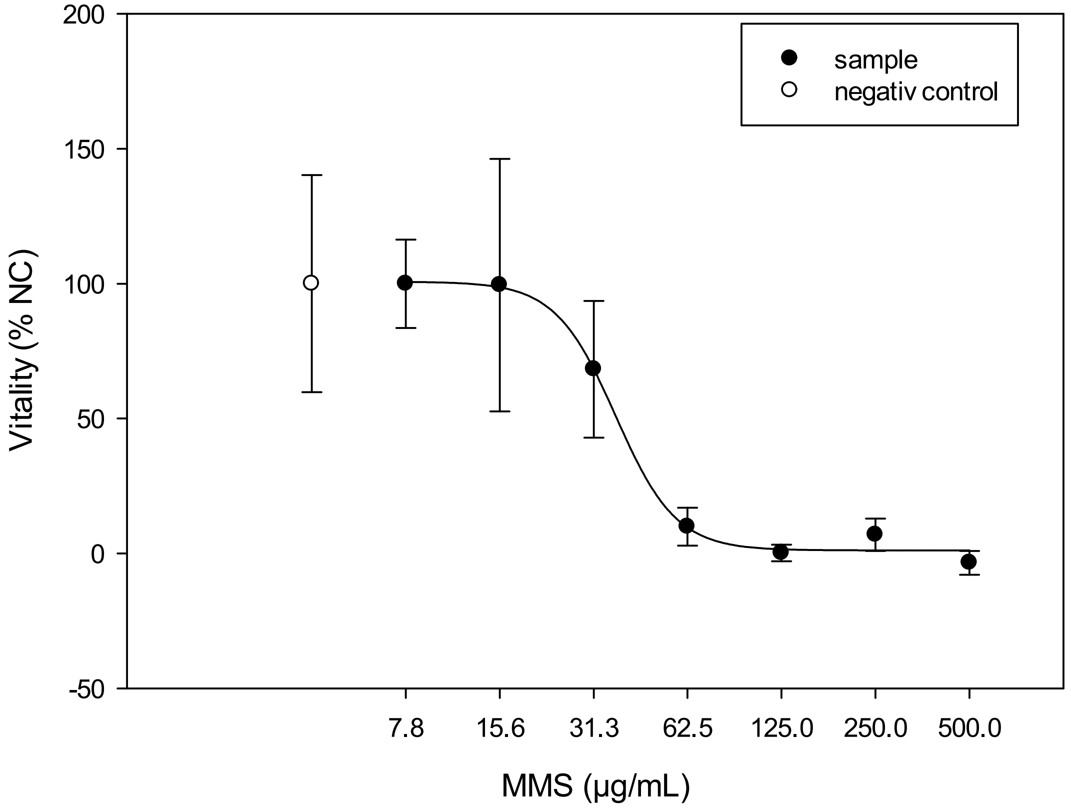

200
150
100
50
0
-50
Vitality (% NC)
sample
negativ control
7.8
15.6
31.3
62.5
125.0
250.0
500.0
MMS (µg/mL)

Figure S3
